# Evaluating Equity-Centered Capacity-Building Programs to Strengthen Implementation Leadership in Southern U.S. HIV Service Organizations

**DOI:** 10.1177/23259582261470700

**Published:** 2026-07-20

**Authors:** Katie A. McCormick, Megan C. Stanton, Katyayani Strohl, Samira B. Ali

**Affiliations:** 1 School of Social Work, 12330 University of Texas at Austin, Austin, TX, USA; 2 Department of Sociology, Anthropology, Criminology and Social Work, 8600 Eastern Connecticut State University, Willimantic, CT, USA; 3 Graduate College of Social Work, 14743 University of Houston, Houston, TX, USA

**Keywords:** implementation leadership, community-based organizations, equity-centered implementation science, relational capacity building, intermediary purveyor organization

## Abstract

**Background:**

In the United States, Southern HIV service organizations (SHSOs) are well-positioned to deliver evidence-based interventions for people living with HIV. However, SHSO leaders often lack the necessary implementation leadership skills. This exploratory study evaluates changes in SHSO leaders’ implementation leadership skills after participation in a capacity-building program.

**Methods:**

Data were collected as part of the pre-/post-program evaluation from SHSO leaders who participated in a capacity-building program.

**Results:**

Participant data (n=98) were analyzed using two-way repeated measures ANOVA to examine changes in Implementation Leadership Scale (ILS) scores pre-/post-program participation. Within-subjects effects showed an overall significant difference in ILS scores between pre/post. Between-subjects effects showed no significant differences between programs on ILS scores. Within-subjects effects showed a significant interaction between time and program type on ILS scores. Results of the multivariate Wilks’ lambda test also showed a significant time-by-program interaction on ILS scores.

**Conclusion:**

Findings underscore the significance of *equity-centered* capacity-building efforts in bolstering the implementation leadership skills of organizational leaders and the need for continued investment in SHSOs.

## Plain Language Summary

The Southern U.S. is disproportionately impacted by the HIV epidemic, resulting in significant health disparities. Southern HIV service organizations (SHSOs) are well-positioned to deliver evidence-based interventions for people living with HIV. However, many of these leaders are not given the tools to implement these interventions. A large university-based Center in the U.S. South offers capacity-building programs to enhance SHSOs' implementation leadership skills. We evaluated whether The Center’s capacity-building programs enhanced the implementation leadership skills of SHSO leaders. The Implementation Leadership Scale (ILS) was used during a pre-/post-program evaluation. SHSO leaders who participated in one of the Center’s capacity-building programs completed the evaluations via online survey. Ninety-eight organizational leaders participated in one of four capacity-building programs. Participants reported low baseline implementation leadership scores, which significantly increased after participating in a capacity-building program. Implementation leadership scores varied by capacity-building program, with the largest increases among participants of a program explicitly focused on leadership development. This study demonstrates the success and importance of equity-centered capacity-building efforts in developing the implementation leadership skills of organizational leaders. Future research is needed to understand what strategies were most effective in facilitating changes in implementation leadership scores.

## Introduction

### HIV Health Inequities in the Southern U.S

As a result of intersecting social and structural factors, extreme health inequities continue to characterize the HIV epidemic in the U.S.^1^ The Southern U.S. has the highest burden of the HIV epidemic compared to all other regions combined.^1^ However, the South is not a monolith; there is notable heterogeneity within the region related to state policy, healthcare infrastructure, and socio-cultural context. For instance, while some states (AR, KY, LA, NC, OK) have adopted and implemented expanded Medicaid coverage that has facilitated improved HIV health outcomes, many states in the region have not (AL, FL, GA, MS, SC, TN, TX), resulting in higher rates of uninsured individuals and greater reliance on underfunded Ryan White and safety-net services.^2^ Similar regional differences are noted in relation to opioid overdose rates and harm reduction policies.^3^

These HIV health inequities are evidenced in the disproportionate burden on historically marginalized groups, particularly Black and Latinx gay, bisexual, and same-gender-loving men and transgender women.^1,4-6^ In addition to disparate rates of diagnosis, these groups also have adverse outcomes at every step of the HIV care continuum, particularly in the South.^4,7^ Social and structural factors, such as HIV stigma, homophobia, racism, poverty, and limited access to health insurance and high-quality health care, are major drivers of these inequities.^4,8^ Further, anti-DEI legislation has been especially prominent in the U.S. South and directly impacted HIV research and education, as well as the quality of services and health care people living with HIV (PLWH) can receive.^9,10^

### Evidence-Based Interventions Are Underutilized

A number of evidence-based individual and structural interventions across the HIV care continuum have been developed to improve health outcomes for PLWH.^11^ At the individual level, biomedical interventions, such as Pre-Exposure Prophylaxis (PrEP), Post-Exposure Prophylaxis (PEP), and anti-retroviral therapy, such as daily pills or long-acting injectables, have been the predominant intervention in HIV prevention and treatment.^12^ Behavioral interventions “prioritize cognitive, affective, and behavioral processes contributing to uptake and engagement in risk reduction, prevention, or treatment”^13^ and include HIV testing programs, motivational interviewing, and brief psychoeducational interventions.^14^ The Undetectable = Untransmittable (U=U) campaign, which emphasizes that a person living with HIV who is on treatment and maintains an undetectable viral load has zero risk of sexually transmitting HIV, is perhaps the most well-known evidence-based structural HIV intervention.^15^ Other structural interventions include, but are not limited to, increasing access to evidence-based tools/services (eg, condoms, sterile syringes), mass media, community mobilization, and capacity building.^16^

Despite the plethora of evidence-based interventions that exist to address the HIV epidemic, inequities persist. Compared to other regions, Southern states have been slower to adopt innovations in HIV prevention and care.^7^ For instance, several Southern states have yet to widely implement antigen combination HIV tests that detect acute HIV infection.^7^ Similarly, the South has been slow to integrate HIV services into routine healthcare and adopt newer prevention technologies (eg, long-acting PrEP).^7^ Further, although technology-based innovations (eg, telehealth) demonstrate promise for addressing geographic barriers in rural areas throughout the South, implementation has been limited due to internet access and regulatory barriers.^7^ Such gaps may be due to systemic factors, such as resource constraints, provider, and policy environments.^7^ These disparities are also due, in part, to implementation barriers such as intervention-community mismatch,^17^ intervention inflexibility,^18^ and client distrust.^19^ As a result, these interventions do not reach the communities that most need them.

### The Need for Implementation Leadership Training

At the same time, a key strength of Southern healthcare systems is the community-based organizations that bridge healthcare and social service systems.^7^ Community-based HIV service organizations are well-positioned to fill these critical gaps in the implementation of evidence-based practices (EBPs) as they are trusted entities that are attuned and responsive to community needs, and also often representative of the communities they serve.^20^ However, these leaders often do not possess skills critical to implementing EBPs.^20^ Yet, this lack of training is not for lack of desire. Recent regional surveys reveal that though staff of SHSOs are not trained in evidence-based approaches (ie, trauma-informed care, gender-affirmative care, harm reduction), they want such training, but funding and resource constraints are persistent barriers.^3,21,22^

Efforts to strengthen the capacity of the HIV workforce have included leadership development programs, such as Next Gen, ELEVATE, and ESCALATE (see https://www.nmac.org/programs/thecenter/). However, these training programs often do not hone in on developing leaders’ *implementation leadership* skills. Implementation leadership refers to “the specific leadership behaviors that are critical for effective [EBP] implementation”.^23^ This training gap can have negative downstream effects for EBP implementation efforts by way of securing funding, motivating staff, and maintaining EBP fidelity.^24-26^ Strong implementation leadership, on the other hand, fosters an optimal organizational climate for EBP implementation, positively influences employee attitudes toward EBPs, and equips staff with specific competencies.^26,27^ Given that organizational leaders are key to successful EBP implementation but may lack the skill set to do so, there is a critical need for training and capacity-building programs that aim to enhance the implementation leadership skills of leaders in community-based HIV service organizations.

### Closing the Implementation Leadership Gap

A capacity-building Center in the U.S. South works to address this gap. This Center is one of four Gilead COMPASS Initiative Coordinating Centers charged with addressing HIV inequities in the South.^28^ The Center provides justice-centered training, coaching, and grant funding to SHSOs. Specifically, its work is guided by equity-centered implementation science, which pays specific attention to how power emerges in the implementation process and shapes implementation outcomes to advance health equity.^29^ Rooted in this, the Center practices a relational approach to capacity building to foster trust and collaboration with organizational partners. The Center offers a diverse set of capacity-building programs that aim to 1) enhance leaders’ knowledge, expertise, and leadership in a particular content area, and 2) enhance learning through the implementation of a grant-funded project. Programs vary by focus area, ranging from trauma-informed care to harm reduction to language justice – collectively referred to as “person-centered care capacity building.” An overview of each program is provided in Table 1.

**Table 1.** The Center’s Capacity-Building Programs

Program name	Focus area	Length	Grant	Structure
LEARN HR	Harm reduction	8-9 months	$10-13k grant	-Bi-weekly cohort learning sessions
				-Monthly individualized coaching sessions
LEARN TILS	Trauma-informed leadership and supervision	5-10 months	$10-20k grant	-Bi-weekly cohort learning sessions
				-Monthly individualized coaching sessions
ACTION	Latinx and Transgender & Gender Non-Conforming Communities	10 months	$25k grant	-Monthly cohort learning sessions
				-Bi-weekly individualized coaching sessions
Transformative Grant Program	Trauma-informed care	18 months	$85k grant	-Monthly individualized coaching sessions

While each program is distinct in its content area of focus and varies by intensity and length of engagement, they share several similarities. First, the programs are delivered virtually via Zoom and co-facilitated by a Center staff member and community-based expert consultant. Second, the programs share three core capacity-building components related to implementation leadership: 1) a two-hour training session focused on how to integrate equity throughout the implementation process using the EPIS framework,^30^ 2) tailored implementation coaching to support implementation action planning, and 3) grant funding to support the implementation of a project related to the program’s focus area.

### The Current Study

Given the Center’s focus on developing the implementation leadership capacity of SHSO leaders and our equity-centered and person-centered approach to doing so, the authors sought to investigate changes in implementation leadership among capacity-building program participants. Specifically, this exploratory study answers the following research questions: (1) What are baseline levels of implementation leadership among participants? (2) Do implementation leadership scores change after participation in a Center capacity-building program? and (3) Are there differential changes in implementation leadership scores by capacity-building program type?

## Methods

### Study Design

This study was reviewed by the University of Houston’s Institutional Review Board (IRB) and approved as exempt (STUDY00002748). Pre-/post-program evaluation data were collected from an online survey of SHSO leaders who participated in one of the aforementioned capacity-building programs. Participants were not provided an incentive as the evaluation data collection was a voluntary component of their participation in a capacity-building program. The reporting of this study conforms to the Revised Standards for Quality Improvement Reporting Excellence (SQUIRE 2.0)^31^ (See Supplementary File 1).

### Eligibility

A total of 98 leaders from 58 SHSOs participated in a capacity-building program between 2021 and 2023. SHSOs were eligible to participate if they met the following inclusion criteria: 1) were located in one of the 12 Southern U.S. states, 2) had a primary organizational purpose of serving people living with HIV, 3) possessed a 501(c)3 status or were fiscally sponsored, and 4) committed at least two staff members to participate in the program. Organizations were excluded if: 1) the organization was located outside of the U.S. South, 2) the organization’s primary purpose was not dedicated to serving people living with HIV, 3) the organization did not have non-profit status or fiscal sponsorship, or 4) the organization was unable to commit two staff members to participate in the given capacity-building program. Leaders self-selected to participate in the program.

### Measures

Quantitative program evaluation data were collected via Qualtrics from participants before each program started (pre-test) and after each program ended (post-test). Pre-/post-tests consisted of six sections: 1) socio-demographic and occupational characteristics, 2) locally developed knowledge, skills, and attitudes measures specific to the program topic area, 3) knowledge, skills, and attitudes measure of the meaningful involvement of PLWH, 4) the Implementation Leadership Scale,^32^ 5) the Collective Leadership Scale,^33^ and 6) participant experience questions (post-test only). Participants were assigned a unique identification number to ensure accurate pre-/post-test response matching and confidentiality. This study specifically examines results from survey section four related to implementation leadership.

The Implementation Leadership Scale (ILS), Supervisor Version is a 12-item scale that measures the strategic leadership for EBP implementation.^32^ There are four subscales: 1) proactive leadership, indicating a leader’s anticipation of and initiative in addressing implementation challenges; 2) knowledgeable leadership, indicating a leader’s deep understanding of the EBP and implementation issues; 3) supportive leadership, indicating a leader’s support for staff’s adoption and use of the EBP; and 4) perseverant leadership, indicating the leader’s consistent support for and responsiveness to EBP implementation challenges and issues. In alignment with the ILS developer’s guidance, individual items were adapted according to the specific focus area of the given program (see Supplementary File 2).

Respondents rate their level of agreement with each statement using a four-point scale ranging from *not at all* (0) to *a very great extent* (4). The overall scale and four subscale scores are calculated by summing item scores and dividing by the number of items in the (sub)scale, creating a subscale score range from 0 to 12 and a total scale score range from 0 to 48. The ILS is a psychometrically sound instrument that has demonstrated excellent internal consistency reliability (*a* = 0.95 - 0.98) and convergent (*a* = 0.62 - 0.75) and discriminant validity (*a* = 0.05 to 0.41). The ILS has been validated for use in numerous service settings, including substance use disorder treatment settings,^23^ acute care settings,^25^ and child welfare settings.^34^

### Statistical Analysis

Data were analyzed using SPSS (version 28). A two-way repeated measures ANOVA (two-way RANOVA) was conducted to examine the differences in matched pre-/post-ILS scores among participants, and whether ILS scores differed based on the capacity-building program. Prior to analysis, data were screened to ensure assumptions of the mixed design were met. Descriptive statistics, to include skewness and kurtosis coefficients, histograms, and normal Q-Q plots, were examined for ILS at pre- and post-for all programs. These demonstrated that the assumption of normality was met on the two measures of ILS across programs. Sphericity was not an issue, as there were only two measures of ILS (Mauchly’s W = 1.000, chi-square_(df = 0)_ = .000, *p* >.). Results of Levene’s test of equality of variances (*p* > .05) and Box’s M test (F = 1.62, *p* > .05) demonstrated that the assumptions of homogeneity of variances and homogeneity of covariance matrices were met. Missing data were handled using listwise deletion to ensure a complete-case analysis, resulting in a final sample size of *n* = 46 for this analysis. Participants, program staff, nor outcome assessors were masked.

To assess potential attrition bias, Fisher’s exact tests were performed to compare baseline socio-demographic characteristics between participants who completed their respective capacity-building program and those lost to follow-up. After excluding missing baseline values, analyses revealed that completion rates did not systematically differ by age group (*n* = 87, *p* = .303), gender (*n* = 88, *p* = .540), sexual orientation (*n* = 88, *p* = .753), ethnicity (*n* = 88, *p* = .628), race (*n* = 88, *p* = .905), or time in the field (*n* = 85, *p* = .470). These results indicate that attrition was balanced across all baseline characteristics.

## Results

### Respondent Characteristics and Descriptive Results

Between 2021 and 2023, a total of 98 SHSO leaders participated in one of the Center’s four capacity-building programs. Participants held a range of roles within their organizations, with most being an Administrative Director (*n* = 27; 28%). The range of time spent in the field of HIV was 0 to 31 years. Additional respondent characteristics are presented in Table 2. Table 3 presents ILS items, means, standard deviations, and reliabilities. Cronbach’s alphas for ILS subscales and the total score ranged from 0.85 - 0.97, demonstrating excellent internal consistency reliability.

**Table 2.** Participant Characteristics, *n* = 98

Variable	​	*n*	%
Participants		98	100
Age	18-26	5	5.1
	27-34	14	14.3
	35-44	30	30.6
	45-54	27	27.6
	55+	11	11.2
Gender	Cisgender Man	26	26.5
	Cisgender Woman	43	43.9
	Non-Binary/Gender Queer	5	5.1
	Transgender Man	2	2.0
	Transgender Woman	8	8.2
	Not Listed	2	2.0
	Prefer Not to Answer/Missing	12	12.2
Sexual Orientation	Bisexual	6	6.1
	Gay	19	19.4
	Lesbian	6	6.1
	Multiple	5	5.1
	Queer	6	6.1
	Straight/Heterosexual	40	40.8
	Another Not Listed	6	6.1
	Prefer Not to Answer/Missing	10	10.2
Ethnicity	Hispanic/Latinx, or of Spanish Origin	23	23.5
	Non-Hispanic/Latinx, or of Spanish Origin	64	65.3
	Missing/Don’t Know	11	11.2
Race	Asian	1	1.0
	Black or African American	51	52.0
	Native Hawaiian/Pacific Islander	1	1.0
	White	21	21.4
	Multi-Racial	5	5.1
	Another Not Listed/Don’t Know	7	7.1
	Prefer Not to Answer/Missing	12	12.2
Role	Administrative Director	27	27.6
	Administrative Support	8	8.2
	Case Manager	3	3.1
	Community Outreach	3	3.1
	Evaluation	1	1.0
	Peer Worker	3	3.2
	Prevention	4	4.1
	Program Director	6	6.1
	Program Manager	6	6.1
	Program Coordinator	8	8.2
	Therapist/Clinician	1	1.0
	Other	17	17.3
	Prefer Not to Answer/Missing	11	11.2
Time in HIV Field	<1 year	7	7.1
	1-4 years	22	22.5
	5-9 years	22	22.5
	10-14 years	13	13.2
	15-19 years	10	10.2
	20-24 years	4	4.0
	25+ years	7	7.1
	Prefer Not to Answer/Missing	13	13.2

**Table 3.** Implementation Leadership Scale (ILS) Subscales and Item Statistics

ILS items, subscales, and totals	Alpha	*Mean*	*SD*
**1. Proactive leadership (n=86)**	.90	2.45	1.05
I can establish clear department standards for the implementation of [topic]. (n=86)	​	2.53	1.13
I can develop a plan to facilitate implementation of [topic]. (n=86)		2.35	1.16
I can remove obstacles to implementation of [topic]. (n=86)		2.45	1.18
**2. Knowledgeable leadership (n=84)**	.96	2.03	1.13
I know what I am talking about when it comes to [topic]. (n=85)	​	2.01	1.19
I am knowledgeable about [topic]. (n=85)		2.11	1.11
I am able to answer staff’s questions about [topic]. (n=85)		1.96	1.24
**3. Supportive leadership (n=79)**	.85	2.98	.99
I can support employee efforts to use [topic]. (n=79)	​	3.19	1.06
I can support employee efforts to learn more about [topic]. (n=85)		3.06	1.14
I can recognize and appreciate employee efforts toward successful implementation of [topic]. (n=85)		2.68	1.18
**4. Perseverant leadership (n=85)**	.97	3.06	.99
I can persevere through the ups and downs of implementing [topic]. (n=85)	​	3.02	1.04
I can carry on through the challenges of implementing [topic]. (n=85)		3.04	1.03
I can react to critical issues regarding implementation of [topic] by openly and effectively addressing the problem(s). (n=85)		3.11	1.00
**Implementation Leadership Scale total (n=79)**	.93	2.60	.86

#### Main Findings

A two-way RANOVA was utilized to examine differences in ILS scores among participants and whether scores differed based on the type of program. Major findings are presented below, and further details are presented in Tables 4 and 5.

**Table 4.** Results of Two-Way Repeated Measures ANOVA, Descriptive Statistics

ILS	Program 1			Program 2			Program 3			*Total*		
	*n*	*Mean*	SE	*n*	*Mean*	SE	*n*	*Mean*	SE	*n*	*Mean*	SE
Pre	23	34.39	9.19	16	28.67	9.63	6	25.75	8.85	45	30.56	9.79
Post	23	43.09	5.07	16	41.33	5.92	6	48.00	8.48	45	44.60	6.94
*Total*	46	38.74	7.13	32	35	7.77	12	36.88	8.66	90	37.58	8.37

*Note.* One program’s sample was too small to compute; SPSS automatically excluded from analysis.

**Table 5.** Results of Two-Way Repeated Measures ANOVA

Source	SS	*df*	MS	F	*p*	*n* ^*2*^ *(partial eta squared)*
Time^a^	3488.12	1	3488.12	83.02	<.001	.664
Program	157.59	2	78.79	.93	.402	.042
Interaction^b^	873.35	2	436.68	10.39	<.001	.331
Subjects	3550.37	42	84.53	​	​	​
Residuals [error(time)]	1764.60	42	42.01	​	​	​

^a^Wilks’ lambda = .336, F_(1,42)_ = 83.02, *p* < .001, *n*^2^ = .664.

^b^Wilks’ lambda = .669, F_(2,42)_ = 10.39, *p* < .001, *n*^2^ = .331.

#### Within-Subjects Effect

The results of the two-way RANOVA tests of within-subjects effects showed an overall significant difference in ILS scores between pre/post (F_(1, 42)_ = 83.02, *p* < .001, *n*^2^ = .664). The results of the multivariate Wilks’ lambda test also showed an overall significant difference between the two mean scores (Wilks’ lambda = .336, F_(1,42)_ = 83.02, *p* < .001, *n*^2^ = .664). The results of the Bonferroni pairwise comparison showed that ILS scores were significantly different across programs. Results showed that post-scores were significantly greater than pre-scores for three out of four programs (*p* < .01). Overall, about 70% of the variance in ILS scores is accounted for by time.

#### Between-Subjects Effect

The results of the two-way RANOVA tests of between-subjects effects showed no significant differences on ILS scores between programs (F_(2,42)_ = .932, *p* > .05), *n*^2^ = .042). Participants across programs reported similar mean scores on ILS at pre-test (LHR: M = 2.87, SE = .77; TGP: M = 2.39, SE = .80; TILS: M = 2.15, SE = .74) and post-test (LHR: M = 3.59, SE = .42; TGP: M = 3.44, SE = .49; TILS: M = 4.0, SE = .71). However, the results of the Bonferroni pairwise comparison showed a significant mean difference between LHR and TILS (*p* < .05). Overall, results showed that program type did not contribute any percentage to the variance in ILS scores.

#### Interaction Effect

The results of the two-way RANOVA tests of within-subjects effects showed a significant interaction between time and program type on ILS scores (F_(2,42)_ = 10.39, *p* < .001, *n*^2^ = .331). Results of the multivariate Wilks’ lambda test also showed a significant time-by-program interaction on ILS scores (Wilks’ lambda = .669, F_(2,42)_ = 10.39, *p* < .001, *n*^2^ = .331).

## Discussion

Implementation leadership plays a central role in the successful implementation of evidence-based practices (EBPs); however, leaders of Southern HIV service organizations (SHSOs) seldom receive explicit, focused training in implementation leadership skills. A large capacity-building Center in the U.S. South builds the implementation leadership capacity of SHSO leaders through robust equity- and person-centered capacity building programming. This exploratory program evaluation study investigated changes in implementation leadership skills among SHSO leaders who participated in a Center capacity-building program.

Participants reported low implementation leadership scores at baseline,^23,25,34^ which significantly increased after participating in a capacity-building program. While the Center’s capacity-building programs were not tested using experimental designs, other tested implementation leadership interventions have reported similar findings.^35,36^ One potential explanation for these significant increases is the structure and content of the capacity-building programs. Though each program focuses on a distinct content area (see Table 1), they share three core implementation leadership capacity-building components. The first of which is a two-hour knowledge and skills-building virtual training session focused on integrating equity throughout the implementation process using the EPIS framework.^30^ In our implementation practice experience, we have found that because many organizations are led by and accountable to the communities they serve, they are often already working with equity and justice in mind, but just lack specific skills in implementation frameworks. Our training on equity-centered implementation aims to bridge this gap by equipping participants with implementation knowledge, skills, and language to, in turn, empower and hold participants accountable to operationalizing in the implementation of their grant-funded projects – the second core capacity-building component. These components are complemented by tailored implementation coaching calls (third capacity-building component) that are facilitated by Center staff and community-based expert consultants, many of whom are PLWH or reflect communities disproportionately impacted by HIV.^37^ These consistent touchpoints between The Center and organizations serve to foster connection, trust, and relationship-building that facilitate learning and application of implementation-related concepts.

Study results also revealed that implementation leadership scores varied by capacity-building program, with the largest increases among *LEARN: Trauma-informed Leadership and Supervision* participants (see McCormick et al., 2023).^38^ This finding stands in contrast to our assumptions that the program with the greatest intensity (as indicated by the “amount” of the program) would demonstrate the largest increases in implementation leadership scores. However, it is also potentially reasonable to conclude that this program demonstrated the largest increases because the core content is fundamentally about leadership, whereas the other programs focus on a substantive content area (eg, trauma-informed care, harm reduction), and leadership development is in service to that substantive content area. This suggests that explicit content on leadership development as an end in itself can bolster changes in perceived implementation leadership.

Findings from this study corroborate our prior qualitative work on person-centered capacity building,^39^ providing quantitative evidence that our programs demonstrate preliminary effectiveness in measurably enhancing SHSO leaders’ implementation leadership capacity. To that end, we suspect that our equity-centered approach to building implementation leadership played an integral role in facilitating such significant changes across all of our programs.^39,40^ In applying equity-centered implementation science in our work with SHSOs, we have found that both person-centered care (eg, trauma-informed care, harm reduction) and person-centered care capacity building are fundamentally about power relationships. Recognizing the power dynamics between us and our partner organizations, we strive to practice a relational approach to capacity building in order to reorganize this power relationship to foster trust, transparency, mutuality, and collaboration.^39,40^ As such, we speculate that three specific strategies were key to enhancing participants’ implementation leadership: 1) facilitating a non-judgmental learning environment that is free of fear associated with lack of knowledge and fosters psychological safety and introspection; 2) trusting partners as experts in their own experiences and empowering them to make decisions that are best for their organization; and 3) being trustworthy through consistent, transparent, and responsive communication practices with partners. Future research is needed to isolate, measure, and test implementation strategies and mechanisms to understand how they facilitate changes in implementation leadership.

### Limitations

The findings of this study should be considered alongside its limitations. In addition to the small sample size, the sample is comprised of participants from SHSOs that were not only interested and willing, but selected, to participate in a capacity-building program; therefore, the sample does not include or account for leaders of SHSOs that did not apply or were not selected to participate. As a result, the study is subject to selection bias, and findings cannot be generalized to other populations or contexts. Use of pre-existing program evaluation data limited analysis to a pre-/post-survey design, and the lack of a control group prevents causal inferences about program effectiveness. Further, pre-/post-surveys relied on individual self-reports of their implementation leadership skills, which may be subject to social desirability bias. Given study design and sample size limitations, future experimental research is needed to test the effects of equity-centered capacity building interventions on individual implementation leadership outcomes; however, this study provides initial insight into the potential impact of such programming for SHSO leaders.

### Conclusion

Increasing implementation leadership skills of leaders in community-based HIV service organizations in the U.S. South is one mechanism for effectively speeding the dissemination and implementation of research evidence into practice. This study underscores the significance of *equity-centered* capacity-building efforts in bolstering the implementation leadership skills of a critical yet marginalized workforce. The Center’s approach to implementation leadership capacity building enhances trust, empowerment, and collaboration – all necessary skills for navigating complex dynamics characteristic of equity-centered HIV health services in the U.S. South. Findings emphasize the importance of continued investment in SHSOs, as such efforts are essential for achieving HIV health justice.

## Supplemental Material

Supplemental Material - Evaluating Equity-Centered Capacity-Building Programs to Strengthen Implementation Leadership in Southern U.S. HIV Service Organizations Supplemental material for Evaluating Equity-Centered Capacity-Building Programs to Strengthen Implementation Leadership in Southern U.S. HIV Service Organizations by Katie A. McCormick, Megan C. Stanton, Katyayani Strohl and Samira B. Ali in Journal of the International Association of Providers of AIDS Care (JIAPAC).

Supplemental Material - Evaluating Equity-Centered Capacity-Building Programs to Strengthen Implementation Leadership in Southern U.S. HIV Service Organizations Supplemental material for Evaluating Equity-Centered Capacity-Building Programs to Strengthen Implementation Leadership in Southern U.S. HIV Service Organizations by Katie A. McCormick, Megan C. Stanton, Katyayani Strohl and Samira B. Ali in Journal of the International Association of Providers of AIDS Care (JIAPAC).

## Data Availability

The data that support the findings of this study are available upon reasonable request from the corresponding author, KM. The data are not publicly available due to their containing information that could compromise the privacy of participants.*
