# Supplementary material for: Evaluating Equity-Centered Capacity-Building Programs to Strengthen Implementation Leadership in Southern U.S. HIV Service Organizations
Source: J Int Assoc Provid AIDS Care. 2026 Jul 20;25:23259582261470700. doi: 10.1177/23259582261470700 (PMC13385600; doi:10.1177/23259582261470700)
Supplement: Supplemental Material - Evaluating Equity-Centered Capacity-Building Programs to Strengthen Implementation Leadership in Southern U.S. HIV Service Organizations [file sj-pdf-1-jia-10.1177_23259582261470700.pdf]

**Supplementary File 2.** Implementation Leadership Scale - Supervisor Version

Please indicate the extent to which you agree with each statement.

| 0<br>Not at all | 1<br>Slight extent | 2<br>Moderate extent | 3<br>Great extent | 4<br>Very great extent |
|---|---|---|---|---|

| | |
|---|---|
| **Proactive** | 0 1 2 3 4 |
| 1. I can develop a plan to facilitate implementation of [topic]. | 0 1 2 3 4 |
| 2. I can remove obstacles to implementation of [topic]. | 0 1 2 3 4 |
| 3. I can establish clear department standards for the implementation of [topic]. | 0 1 2 3 4 |
| **Knowledgeable** | 0 1 2 3 4 |
| 4. I am knowledgeable about [topic]. | 0 1 2 3 4 |
| 5. I am able to answer staff's questions about [topic]. | 0 1 2 3 4 |
| 6. I know what I am talking about when it comes to [topic]. | 0 1 2 3 4 |
| **Supportive** | 0 1 2 3 4 |
| 7. I can recognize and appreciate employee efforts toward successful implementation of [topic]. | 0 1 2 3 4 |
| 8. I can support employee efforts to learn more about [topic]. | 0 1 2 3 4 |
| 9. I can support employee efforts to use [topic]. | 0 1 2 3 4 |
| **Perseverant** | 0 1 2 3 4 |
| 10. I can persevere through the ups and downs of implementing [topic]. | 0 1 2 3 4 |
| 11. I can carry on through the challenges of implementing [topic]. | 0 1 2 3 4 |
| 12. I can react to critical issues regarding implementation of [topic] by openly and effectively addressing the problem(s). | 0 1 2 3 4 |

**Notes*. Adapted from Aarons et al., 2014.[32] In alignment with the developer's guidance, each item was adapted according to the specific focus area of the given program: harm reduction; trauma-informed leadership and supervision; Latinx, transgender, and non-conforming communities; trauma-informed care (see Table 1).
